# Effects of dietary supplementation of chromium methionine chelate on growth performance, oxidative stress, hematological indices, and carcass traits of broiler chickens

**DOI:** 10.1007/s11250-022-03260-1

**Published:** 2022-08-16

**Authors:** Ibrahim M. I. Youssef, Ibrahim M. I. Abdo, Hassan F. A. Elsukkary, Magdy F. El-Kady, Magdy Elsayed

**Affiliations:** 1 Department of Nutrition and Clinical Nutrition, Faculty of Veterinary Medicine, Beni-Suef University, Beni-Suef, 62511 Egypt; 2 United BioMed Company for Feed Premixes and Chemicals, Cairo, Egypt; 3 Department of Poultry Diseases, Faculty of Veterinary Medicine, Beni-Suef University, Beni-Suef, 62511 Egypt; 4 Department of Infectious Diseases, Faculty of Veterinary Medicine, Cairo University, Giza, 12211 Egypt

**Keywords:** Chelated chromium, Broilers, Productive performance, Antioxidant activity, Carcass traits

## Abstract

This study was conducted to evaluate the dietary effects of chromium methionine (Cr-Meth) chelate on growth performance, oxidative stress parameters, blood biochemistry, and carcass traits of broiler chickens. An experiment was conducted on 34,000 1-day-old straight-run broiler chicks (Indian River; 42.0 ± 0.03) at a commercial farm. The chicks were divided randomly into 3 groups; the first group contained 17,000 birds, which used as a control, whereas the second and third groups consisted of 7000 and 10,000 birds, respectively, with 5 replicates per group. A completely randomized design was used. The birds were fed the experimental diets containing graded levels of Cr-Meth chelate: 0 (control), 50, and 100 g/ton. This compound consisted of chromium (0.4%) chelated with methionine, and it supply the diets with 200 and 400 ppb Cr for the used levels of 50 and 100 g/ton feed, respectively. Growth performance indices (body weight, body weight gain, feed intake, and feed conversion ratio) were measured throughout the experiment. At the end of experiment, 10 birds per treatment were slaughtered, and the carcass yield with relative weight of the internal organs was determined. Also, blood samples were taken and analyzed for glutathione peroxidase activity, malondialdehyde, ALT, AST, total protein, albumin, glucose, urea, creatinine, triglycerides, and total cholesterol. It was found that Cr-Meth improved the body weight, weight gain, feed intake, and feed conversion ratio of broilers. Moreover, it reduced the mortality rate of birds. The chelated chromium can alleviate the oxidative status of birds by increasing the plasma glutathione peroxidase activity and reducing the serum malondialdehyde level. It was observed that the effects of 100 g/ton Cr-Meth chelate on performance indices, mortality rate, and oxidative stress parameters were better than that of 50 g/ton inclusion rate. Supplementation of Cr-Meth increased the total protein level, but reduced the glucose, total cholesterol, and triglyceride concentrations in the blood serum. In addition, it increased the carcass yield and reduced the abdominal fat percentage of the birds’ carcass. Therefore, chromium can be included in diets of broilers at a rate of 200 to 400 ppb, and the higher concentration was more effective than the lower one. So, it can be recommended to use Cr-Meth chelate in broiler diets at 100 g/ton to improve the productive performance and reduce the oxidative stress of birds.

## Introduction

Dietary chromium supplementation has been reported to increase the growth rate and feed efficiency, and improve the meat yield and carcass quality with reduced carcass fat in broilers (Toghyani et al., 2006; Samanta et al., 2008). Its beneficial effects seem to be greater under stress condition (Borgs and Mallard, 1998). The role of chromium in carbohydrate, protein, and lipid metabolism is well documented. Glucose tolerance factor in the body, which is activated by chromium, is responsible to make the metabolic function of insulin more effective (Arif et al., 2019). Chromium supplementation can decrease the carcass fat percentage and also decreased the plasma cholesterol level in broilers (Kim et al., 1996). Total cholesterol and triglyceride proportions of breast and thigh meat were reduced in broilers supplemented with organic chromium at rates of 200 ppb and 400 ppb (Gursoy, 2000). Supplemental chromium in broilers’ diets was found to enhance the insulin activity and increase the glucose uptake by the cells. It has been reported that supplemental chromium has raised the total serum protein and insulin concentrations, whereas reducing the cholesterol blood levels (Gursoy, 2000; Arif et al., 2019). Moreover, supplemental trivalent organic chromium improved the growth, breast meat yield, and carcass quality in broilers (Habibian et al., 2013). It is used in diets of poultry and animals to enhance the performance and productivity (Debski et al., 2004). Tawfeek et al. (2014) reported that 500 ppb of chromium can reduce the heat stress and improve the performance of broilers.

Different chromium forms (organic and inorganic) have different rates of absorption (Hamidi et al., 2016). Inorganic chromium compounds are poorly absorbed in humans and animals, and its absorption ranges from 0.4 to 3% or less, regardless of dose and dietary Cr status (Anderson et al., 1983). However, bioavailability of organic chromium is about 25–30% (Mowat, 1997). Dietary chromium supplementation has been suggested to have beneficial effects in animals, especially when under stress. During the last two decades, the effects of supplemental chromium on a variety of variables in poultry have a great attention (White and Vincent, 2019). It is assumed that organic chromium, especially Cr methionine chelate, has marked positive effects on productive performance, and can increase the resistance of poultry against oxidative stress. Therefore, the present study was conducted to evaluate the effects of dietary chromium methionine chelate on growth performance, oxidative stress parameters, blood biochemistry, and carcass traits of broiler chickens.

## Materials and methods

### Preparation of chromium methionine chelate

The synthesis of chromium methionine chelate was done at United BioMed Company for feed premixes and chemicals (Cairo, Egypt), by using the procedure of Tang et al. (2013). The used DL-methionine (feed grade; 99%) was produced by Evonik Industries (Essen, Germany), whereas chromium(III) chloride hexahydrate (97%) was obtained from Scharlau Chemicals (Barcelona, Spain). Chromium(III) chloride hexahydrate 97% (82.194 g; 0.3 mol) and aqueous ethanol (50%; 1500 mL) were heated to 85 °C in a refluxed three-neck flask, equipped with a thermometer and a dropping funnel, on a magnetic stirrer hot plate. A mixture of 1.2 mol methionine and 1.2 mol sodium hydroxide and aqueous ethanol (50%; 600 mL) was stirred at the same temperature until completely dissolved. Afterwards, the mixture was cooled to 25 °C, and then, it was added to the three-neck flask dropwise, and stirred at 80 °C for 2.5 h. The reaction mixture was cooled to 25 °C, and then, the solid was filtered, washed with cool water, and dried in an oven at 100 °C. The obtained product was checked by using FTIR (Fourier transform infrared) assay, according to Ashmead (2012). The FTIR spectrum was recorded on a Bruker FTIR spectrometer (Vector 22) in the range of 4000 − 400 cm^−1^. The Cr-Meth product was formulated to contain 0.4% Cr and 3.44% methionine.

### Birds, husbandry, and feeding

This experiment was conducted on 34,000 1-day-old straight-run broiler chicks (Indian River) at a commercial farm. The chicks were divided randomly into 3 groups; the first group contained 17,000 birds, which used as a control, whereas the second and third groups consisted of 7000 and 10,000 birds, respectively. Each group was subdivided into 5 replicates, with 3400, 1400, and 2000 birds per replicate in the first, second, and third treatments, respectively. The number of birds was not equal in each group due to the difference in pen sizes inside the farm; besides, it was planned that the largest number was the control while the smaller numbers were the experimental treatments. The birds were fed the experimental diets containing graded levels of chromium methionine (Cr-Meth) chelate: 0 (control), 50, and 100 g/ton. This compound consisted of chromium (0.4%) chelated with methionine (3.44%), and it supplies the diets with 200 and 400 ppb Cr for the used levels of 50 and 100 g/ton inclusion rate, respectively. The chicks were fed a starter diet from the beginning of the experiment and up to day 10 of age, and then a grower one until day 24, followed by a finisher diet till the end of the experiment at day 37. The diets were offered to the birds in the form of pellets. The diets were formulated to satisfy the nutrient requirements of the birds based on the nutritional breed specifications. The chelated Cr-Meth was added to the basal diets substituting equal amounts of the yellow corn ingredient. The added DL-methionine to the diets was adjusted according to the methionine amount supplied by the Cr-Meth compound. The diets were analyzed for crude protein, ether extract, crude fiber, calcium, and total phosphorus according to AOAC (2005) procedures, while the other nutrients were calculated by using the feed composition tables of NRC (1994) for poultry. The ingredients and chemical composition of the basal diets are shown in Table 1. The energy enzyme and phytase were included in the diets to break down the anti-nutritional factors (non-starch polysaccharides and phytates, respectively) found in the cereal grains and soybean meal. Moreover, the nutritional matrix values of these enzymes were used in formulation of the diets; the energy enzyme provides 85.0 kcal ME/kg, and phytase releases 0.11% available phosphorus, according to the produced companies. The experiment lasted for 37 days. The ambient temperature was gradually reduced from 33 °C at day 1 of age to about 24 °C at the end of the experiment. The relative humidity should be within 50 and 65%. The light during the first 4 days of age was provided for 24 h continuously. From day 5 of age onwards, the daily light was reduced to 18 h. Feed was offered in automatic feeders ad libitum. Fresh water was continuously supplied by nipple drinkers.

**Table 1:** Ingredients and chemical composition (%) of the basal starter, grower, and finisher diets fed to broilers (as fed)

	Starter (0–10 days)	Grower (11–24 days)	Finisher (25–37 days)
Ingredients (%)			
Yellow corn, ground	54.92	58.57	63.70
Soybean meal, 46% CP	36.51	31.31	25.21
Corn gluten meal, 60% CP	3.30	4.32	5.0
Limestone	1.85	1.78	1.57
Monocalcium phosphate	1.15	1.0	0.90
Soybean oil	0.73	1.55	2.14
Vitamin and mineral mixture*	0.3	0.3	0.3
Salt (NaCl)	0.27	0.27	0.27
L-lysine HCl	0.23	0.21	0.25
DL-methionine	0.17	0.14	0.12
Sodium bicarbonate	0.15	0.15	0.15
Choline chloride	0.10	0.10	0.10
Antimycotoxins	0.10	0.10	0.10
Threonine	0.06	0.04	0.03
Energy enzyme	0.05	0.05	0.05
Anticoccidia	0.05	0.05	0.05
Anticlostridia	0.05	0.05	0.05
Phytase	0.01	0.01	0.01
Chemical composition			
Metabolizable energy (kcal/kg)	3000.5	3103.5	3202.0
Crude protein (%)	22.98	21.35	19.40
Ether extract (%)	3.05	3.88	4.56
Crude fiber (%)	2.59	2.61	2.50
Methionine (%)	0.56	0.52	0.48
Methionine + cystine (%)	0.96	0.89	0.82
Lysine (%)	1.44	1.29	1.16
Threonine (%)	0.97	0.88	0.79
Calcium (%)	1.01	0.90	0.80
Total phosphorus (%)	0.63	0.60	0.55
Available phosphorus (%)	0.48	0.44	0.41
Sodium (%)	0.16	0.16	0.16

^*^Poultry vitamin and mineral premix: each 3 kg contains vit. A, 12,000,000 IU; vit. D_3_, 2,000,000 IU; vit. E, 10,000 mg; vit. K_3_, 2000 mg; vit. B_1_, 1000 mg, vit.B_2_, 5000 mg; vit.B_6_, 1500 mg; vit. B_12_, 10 mg; biotin, 50 mg; pantothenic acid, 10,000 mg; nicotinic acid, 30,000 mg; folic acid, 1000 mg; Mn, 60,000 mg; Zn, 50,000 mg; Fe, 30,000 mg; Cu, 10,000 mg; I, 1000 mg; Se, 100 mg; Co, 100 mg; and calcium carbonate up to 3 kg

### Growth performance

The birds were weighed at the start and end of the experiment, and the weight gain was determined. Weighed amounts of feed were offered daily, and the feed intake was calculated. Afterwards, the feed conversion ratio (FCR), corrected for dead birds, was calculated by dividing the feed intake by the weight gain. The mortality rate was recorded daily throughout the experiment.

For calculation of the body weight gain per broiler chicken, the following formula was used:

Average weight gain per bird for the feeding period → *F* – *S* (corrected by weight gain of died or culled chickens).

*F*: average weight of the live birds in the pen at the end of experiment.

*S*: average weight of the live birds in the pen at the start of experiment.

The feed intake (corrected for dispersed feed) was calculated by using the following formula:

$$\mathrm{Feed}\;\mathrm{intake}\;\mathrm{per}\;\mathrm{period}=\frac{\mathrm{total}\;\mathrm{feed}\;\mathrm{consumed}\;\mathrm{per}\;\mathrm{pen}}{\left(\mathrm{number}\;\mathrm{of}\;\mathrm{surviving}\;\mathrm{birds}\;\times\;\mathrm{days}\;\mathrm{of}\;\mathrm{the}\;\mathrm{period}\right)+\mathrm{days}\;\mathrm{of}\;\mathrm{died}\;\mathrm{birds}\;\mathrm{alive}}$$

The feed conversion ratio was estimated by using the following formula:

$$\mathrm{Feed}\;\mathrm{conversion}\;\mathrm{per}\;\mathrm{period}=\frac{\mathrm{total}\;\mathrm{feed}\;\mathrm{consumed}\;\mathrm{for}\;\mathrm{the}\;\mathrm{period}\;\mathrm{in}\;\mathrm{each}\;\mathrm{replicate}}{\mathrm{total}\;\mathrm{weight}\;\mathrm{gain}\;\mathrm{for}\;\mathrm{the}\;\mathrm{period}\;\left(\mathrm{with}\;\mathrm{weight}\;\mathrm{gain}\;\mathrm{of}\;\mathrm{died}\;\mathrm{or}\;\mathrm{culled}\;\mathrm{chikens}\right)}$$

### Blood parameters

At the end of the experiment, blood samples were collected from 10 chickens in each group (2 birds per replicate). Five milliliters of blood was taken from each bird in a sterile plastic syringe. Two milliliters of the blood samples was collected in sterile heparinized centrifuge tubes. These samples were centrifuged at 3000 r.p.m. for 15 min for separation of blood plasma. The other 3 mL of the blood samples was collected in clean centrifuge tubes and left at room temperature for 30 min to clot, and then centrifuged also at 3000 r.p.m. for 15 min, for separation of blood serum. Collected plasma and serum were stored in a deep freezer at − 20 °C until the chemical analyses. At the time of analysis, the plasma samples were used for determination of glutathione peroxidase (GPx) according to Mates et al. (2000) method, whereas the serum samples were used for measurement of malondialdehyde (MDA), alanine aminotransferase (ALT), aspartate aminotransferase (AST), glucose, total protein, albumin, total triglycerides, total cholesterol, creatinine, and urea by using commercial test kits (Diamond Diagnostic Company, Egypt). Globulin was determined by subtracting albumin from total protein. Serum ALT and AST, creatinine, and urea were measured to evaluate the effect of chelated chromium levels on liver and kidney functions, respectively, while the other serum parameters were used as a tool to detect the physiological alterations, fitness of the birds, and carbohydrate, protein, and lipid metabolism.

### Carcass characteristics

Ten birds from each group (2 birds/replicate), close to the average live body weight, were selected at the end of the experiment. Birds were weighed to the nearest gram, and slaughtered by the neck cutting (Halal method). The carcass without giblets was weighed, expressed as a percentage of its live body weight, and considered as the carcass yield. In addition, the weight of the breast, liver (without gall bladder), gizzard, proventriculus, heart, spleen, and visible fat (around the viscera, gizzard, and subcutaneously) were determined, and its relation to the live body weight of the birds, in percentages, was calculated.

### Statistical analyses

Statistical analysis of the experimental data was performed using SPSS statistical program (IBM, version 22, Chicago, USA, 2013) to determine the variable differences between the treatments. The data were analysed using the general linear model (GLM) procedure for analysis of variance. The results were subjected to one-way ANOVA test accompanied by Duncan’s multiple range test to determine the differences between the treatments. The statistical model was as follows: *Y*_*ij*_ = *µ* + *T*_*i*_ + *E*_*ij*_, where *Y*_*ij*_ = the observation *ij*, *µ* = the overall mean, *T*_*i*_ = the effect due to treatment *i*, and *E*_*ij*_ = the experimental error. Polynomial contrasts were used to test linear and quadratic effects of Cr-Met chelate supplementation. The results are presented as means ± SEM. Probability values less than 0.05 (*P* < 0.05) were considered significant.

## Results

**Fig. 1:**
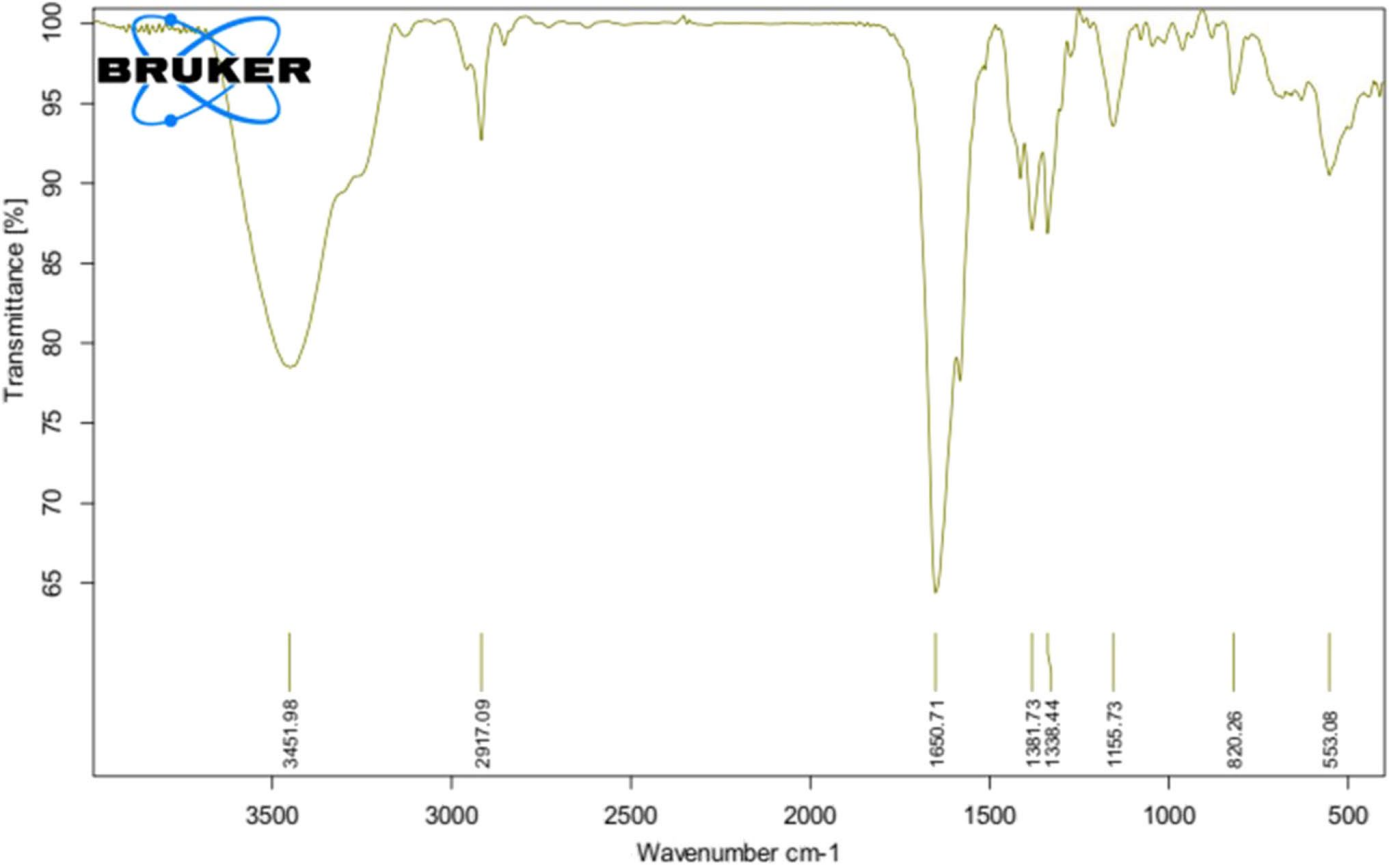
FTIR (Fourier transform infrared) analysis of chromium methionine chelate

The result of FTIR analysis, which proves the chelation process of Cr with methionine, is shown in Fig. 1. The IR spectrum of Cr(III) methionine chelate showed a sharp very strong band at 1650 cm^−1^ corresponding to asymmetric stretching of COO^−^. The IR spectrum exhibited disappearance of –NH_3_^−^ stretching band at 2109 cm^−1^, which appeared in methionine. The IR spectrum confirmed the chelation by N–H stretching broad bands at 3451 cm^−1^ and at 3239 cm^−1^.

The effect of Cr-Meth on growth performance of broilers is presented in Table 2. The body weight and weight gain of birds were significantly (*P* < 0.05) increased by increasing the level of Cr-Meth chelate in the diets. It was noticed that the weight and weight gain were higher (*P* < 0.05) in birds fed diets containing 100 g/t Cr-Meth than in 50 g/t supplemented group. The feed intake was increased in birds fed Cr-supplemented diets than the control, which was significantly (*P* < 0.05) higher in 100 g/t Cr-Meth treatment. Moreover, the feed conversion ratio of the chickens was decreased (*P* < 0.05) in Cr-supplemented groups when compared to the control, resulting in an improvement in feed utilization efficiency by the dietary chromium supply. Moreover, the FCR did not differ (*P* > 0.05) between 50 and 100 g/t Cr-Meth treatments. The mortality rate was subsided by increasing the inclusion rate of chelated chromium in the diets, which was significantly (*P* < 0.05) lower in case of the higher level of chromium inclusion (100 g/t). It was observed that the effect of Cr-Meth chelate on body weight, weight gain, and feed intake was linear (*P* < 0.05), whereas it was quadratic (*P* < 0.05) in case of FCR and mortality rate.

**Table 2:** Growth performance of broilers fed diets containing different levels of chromium methionine chelate

	Cr-Meth chelate level (g/ton feed)			SEM	*P* value		
	0 (Control)	50	100		Treatment	Linear	Quadratic
Initial body weight (g/bird)	42.01	43.0	41.02	0.03	0.315	0.476	0.654
Final body weight (g/bird)	2185.6 ^c^	2251.1 ^b^	2302.5 ^a^	19.02	0.010	0.023	0.125
Feed intake (g/bird)	3418.1 ^b^	3449.3 ^ab^	3510.6 ^a^	28.18	0.043	0.044	0.240
Weight gain (g/bird)	2143.6 ^c^	2208.1 ^b^	2261.5 ^a^	17.50	0.012	0.020	0.356
FCR	1.59 ^a^	1.56 ^b^	1.55 ^b^	0.01	0.036	0.267	0.039
Mortality rate (%)	3.53 ^a^	3.50 ^a^	3.15 ^b^	0.02	0.041	0.213	0.047

Means within the same row with different letters are significantly different (*P* < 0.05)

The effect of chromium methionine chelate on oxidative stress parameters is shown in Table 3. The plasma glutathione peroxidase (GPx) activity, as one of the antioxidant enzymes, was increased (*P* < 0.05) by supplementation of Cr-Meth chelate to the diet, and no significant (*P* > 0.05) difference in the enzyme activity was observed between 50 and 100 g/t chelated chromium. In contrast, the serum malondialdehyde (MDA) level, as an oxidative stress indicator, decreased significantly (*P* < 0.05) by increasing the inclusion rate of Cr-Meth chelate. The effect of Cr-Meth chelate was linear and quadratic (*P* < 0.05) in case of GPx and MDA.

**Table 3:** Blood parameters of broilers fed diets containing different levels of chromium methionine chelate

	Cr-Meth chelate level (g/ton feed)			SEM	*P* value		
	0 (Control)	50	100		Treatment	Linear	Quadratic
Plasma							
GPx (mU/mL)	2.16 ^b^	2.53 ^a^	2.43 ^a^	0.05	< 0.001	0.001	0.001
Serum							
MDA (nmol/mL)	2.00 ^a^	1.78 ^b^	1.70 ^c^	0.04	< 0.001	0.001	0.030
ALT (U/L)	36.98	33.07	39.15	1.35	0.136	0.239	0.015
AST (U/L)	52.14	45.22	53.51	1.65	0.056	0.373	0.004
Total protein (g/dL)	4.66 ^c^	5.28 ^b^	5.72 ^a^	0.11	< 0.001	0.001	0.487
Albumin (g/dL)	3.06 ^a^	2.71 ^b^	3.01 ^a^	0.04	< 0.001	0.373	0.001
Globulin (g/dL)	1.60 ^b^	2.56 ^a^	2.71 ^a^	0.12	< 0.001	0.001	0.007
Glucose (mg/dL)	191.46 ^a^	173.93 ^b^	161.69 ^c^	2.93	< 0.001	0.001	0.001
Urea (mg/dL)	31.05	29.65	30.63	0.40	0.125	0.444	0.001
Creatinine (mg/dL)	1.72	1.76	1.78	0.02	0.201	0.001	0.240
Triglycerides (mg/dL)	82.29 ^a^	70.18 ^b^	75.14 ^b^	1.03	< 0.001	0.001	0.001
Total cholesterol (mg/dL)	143.85 ^a^	136.43 ^b^	127.64 ^c^	1.60	< 0.001	0.001	0.611

Means within the same row with different letters are significantly different (*P* < 0.05)

*GPx* glutathione peroxidase, *MDA* malondialdehyde, *ALT* alanine aminotransferase, *AST* aspartate aminotransferase

There was no significant (*P* > 0.05) difference in serum ALT and AST activities among the treatments (Table 3). The same results were observed for creatinine and urea concentrations. This indicates that chromium levels, in this study, had no effect on liver or kidney functions. Moreover, the chromium supplemented groups showed an increase (*P* < 0.05) in total protein and globulin levels, and a decrease (*P* < 0.05) in glucose, total triglycerides, and total cholesterol concentrations in the serum. It was noticed that the total protein, glucose, and total cholesterol were affected by the level of chromium in the diets, as the protein increased, while glucose and cholesterol decreased, by increasing the rate of dietary chromium inclusion. The effect of Cr-Meth chelate was linear and quadratic (*P* < 0.05) in case of globulin, glucose, and triglycerides, whereas it was linear (*P* < 0.05) in total protein, creatinine, and total cholesterol, and quadratic (*P* < 0.05) in ALT, AST, albumin, and urea.

Concerning the carcass traits of birds, supplementation of Cr-Meth chelate was found to increase (*P* < 0.05) the carcass yield (Table 4). However, there were no significant (*P* > 0.05) differences in breast meat yield or internal organs weight among the treatments. Nevertheless, the abdominal fat percentage was significantly (*P* < 0.05) decreased by the chromium supplementation when compared to the control group (about 1.03 vs. 1.38%). Moreover, there was no difference (*P* > 0.05) in the abdominal fat % between 50 and 100 g/t Cr-Meth treatments. The effect of Cr-Meth chelate on carcass yield was linear (*P* < 0.05), but quadratic (*P* < 0.05) in case of abdominal fat.

**Table 4:** Carcass characteristics (% of BW) of broilers fed diets containing different levels of chromium methionine chelate

	Cr-Meth chelate level (g/ton feed)			SEM	*P* value		
	0 (Control)	50	100		Treatment	Linear	Quadratic
Carcass yield	72.82 ^b^	73.90 ^a^	74.41 ^a^	0.53	0.032	0.047	0.299
Breast yield	27.76	28.45	28.50	0.41	0.155	0.814	0.858
Liver	2.81	2.58	2.57	0.08	0.407	0.236	0.549
Heart	0.51	0.53	0.51	0.02	0.418	0.937	0.539
Spleen	0.15	0.10	0.13	0.01	0.368	0.657	0.187
Proventriculus	0.55	0.45	0.52	0.04	0.539	0.742	0.297
Gizzard	2.16	2.14	2.06	0.09	0.309	0.687	0.882
Visible fat	1.38 ^a^	0.96 ^b^	1.10 ^b^	0.08	0.025	0.343	0.036

Means within the same row with different letters are significantly different (*P* < 0.05)

## Discussion

The tested level of Cr-Meth chelate (50–100 g/ton diet) in this study was used to supply the diet with 200–400 ppb Cr. This level was assumed to produce a marked positive effect on the productive performance, and do not have any harmful or toxic impact on the health of birds. Moreover, this dose of chelated Cr was chosen to be more economical when added to poultry diets.

### Growth performance

In the present study, chelated Cr-Meth enhanced the body weight and weight gain of chickens. The effect of chromium on these performance parameters could be due to its function in metabolism of dietary nutrients. The main role of chromium in metabolism is in the area of improving glucose uptake by living tissues. Also, chromium was found to stimulate certain enzymes and stabilizes proteins and nucleic acids as well as it can increase the synthesis of fats in adipose tissues (Amata, 2013). Arif et al. (2019) reported that chromium propionate supplementation improved the weight gain of broilers, and the better performance and weight gain can be achieved when Cr propionate is added at the rate of 400 ppb to the diets. Moreover, it was found that the birds significantly gained higher body weights and gains than the control with the addition of 0.50 mg/kg Cr as chromium picolinate (Tawfeek et al., 2014). The same effect was reported by Naghieh et al. (2010) with 600 µg/kg Cr nicotinate, by Noori et al. (2012) with Cr methionine from 200 to 800 ppb, and by Toghyani et al. (2012) with 1500 ppb organic or inorganic Cr. Recently, Feng et al. (2021) found that Cr picolinate supplementation had a positive effect on the growth performance of broilers. It is well recognized that chromium is essential for proper insulin functioning and also needed for normal protein, fat, and carbohydrate metabolism (Chowdhury et al., 2003). Moreover, greater uptake of glucose to the muscle and adipose tissues constitutes anabolism which increases serum growth factor concentrations (insulin growth factor-1) that enhance the protein formation in broilers (Mohammed et al., 2014). Additionally, efficacy of chromium in the organic complex is superior to Cr in inorganic form (Suksombat and Kanchanatawee, 2005; Sahin et al., 2010). The effect of Cr-Meth chelate could be related to the fact that it is a stable complex structure, and has greater absorbability and bioavailability (Anderson et al., 2004; Zhang and Kim, 2014).

It was found that the Cr-Meth chelate increased the feed intake of broilers. This could be due to an increased appetite by the chromium, and consequently, the birds consumed more diets supplemented with Cr. The same results were reported by the previous studies (Sahin et al., 2003; Toghyani et al., 2006; Naghieh et al., 2010). On the contrary, Tawfeek et al. (2014) observed a reduced feed intake with the dietary Cr supplementation. However, other studies reported no significant effect of Cr supplementation on the feed intake (Anandhi et al., 2006; Jackson et al., 2008).

The positive effect of chromium supplement on the feed conversion rate might be attributed to the improvement in nutrient digestibility and efficiency of its use. In addition, reduction in FCR could be due to the maximum utilization of glucose from blood. Moreover, chromium supplementation exhibited an increased insulin activity, resulting in more glucose absorption and amino acid utilization to produce energy, muscle development, and fat formation (Anderson, 1994). This result is supported by that of the previous research (Sahin et al., 2003; Jackson et al., 2008; Adebiyi and Makanjuola, 2011). Moreover, Arif et al. (2019) found that chromium propionate supplementation improved the feed conversion ratio of broiler chickens. Because Cr could protect the pancreatic tissue against oxidative damage, it may help the pancreas to function properly including secretions of digestive enzymes, thus improving the digestibility of nutrients and consequently the growth performance. However, previous studies revealed no significant effects of Cr on the feed conversion under high temperatures (Toghyani et al., 2006; Naghieh et al., 2010; Noori et al., 2012).

In the present study, supplementation of Cr-Meth chelate was found to decrease the mortality rate throughout the experiment. This finding could be due to the role of Cr as an antioxidant, resulting in protection of the body cells from the oxidative damage (Haq et al., 2016).

### Blood parameters

Dietary Cr-Meth supplementation increased the plasma GPx activity in the broiler chickens, but decreased the serum malondialdehyde concentration. These findings are supported by the results of Tawfeek et al. (2014). The obtained results indicate that the Cr has an antioxidant function, which protects the tissues from damage. Chromium supplementation can increase the superoxide dismutase activity which decreases the oxidative stress and lipid peroxidation, consequently reducing the MDA levels which is a good indicator for stress (Rao et al., 2016; Sahin et al., 2017).

The Cr-Met chelate was found to increase the total serum protein level. This is related to its activity in increasing the globulin levels. In addition, this could be due to enhancing the metabolism of protein and increasing the nutrient digestibility by Cr. However, the Cr supplementation decreased the glucose, cholesterol, and triglyceride concentrations in the serum of chickens. Sahin et al. (2003) reported that Cr resulted in an increased total serum protein but decreased glucose and cholesterol concentrations in broilers. In contrast, other studies reported that the addition of Cr has no influence on these blood parameters (Adebiyi and Makanjuola, 2011; Toghyani et al., 2012). The lower serum glucose level in the Cr-supplemented birds was perhaps indicative of increased turnover rate and utilization of glucose in the body tissues via increasing the activity of insulin by chromium (Tawfeek et al., 2014; Rajalekshmi et al., 2014; Arif et al., 2019). Moreover, the reduction in serum cholesterol and triglyceride levels could be attributed to the role of Cr in lipid metabolism (Hamidi et al., 2016).

### Carcass characteristics

In the present study, the dietary Cr-Meth supplementation increased the carcass yield and reduced the abdominal fat. However, there was no effect of Cr on breast meat yield or relative weight of the internal organs. The increase in the carcass yield by Cr addition was noticed to coincide with the increase in the body weight of birds. Habibian et al. (2013) found that supplemental organic Cr improved the carcass quality of broilers. Moreover, previous studies reported that the abdominal fat was reduced by the addition of Cr (Gursoy, 2000; Sahin et al., 2003; Toghyani et al., 2012; Tawfeek et al., 2014). This could be related to the activity of Cr in metabolism and breakdown of lipids. Moreover, Cr improves insulin activity, resulting in enhancements in carbohydrate, protein, and lipid metabolism, as reflected by reduced non-esterified fatty acids, fastened serum triglyceride removal, and uptake of glucose for lipogenesis in the liver (Lien et al., 1999). The obtained results of internal body organs indicate that the used dietary chromium levels had no any detrimental effects on the liver or other tissues.

## Conclusion

The obtained results indicate that Cr-Meth chelate improved the growth performance parameters (body weight, weight gain, feed intake, and feed conversion efficiency) of broiler chickens. Moreover, it can alleviate the oxidative status of birds by increasing the plasma glutathione peroxidase activity and reducing the serum malondialdehyde level, resulting in a lower mortality rate in the broilers. Supplementation of Cr-Meth increased the total protein, but reduced the glucose, total cholesterol, and triglyceride concentrations in the blood serum. In addition, it increased the carcass yield and reduced the abdominal fat percentage of the birds’ carcass. Therefore, chromium can be included in diets of broilers at a rate of 200 to 400 ppb, and the higher concentration was more effective than the lower one. So, it can be recommended to use chromium methionine chelate in broiler diets at 100 g/ton to improve the productive performance and reduce the oxidative stress of birds.

## Data Availability

The data of this study are available from the corresponding author upon reasonable request.
